# Rëâdīńg wõrdš wîth ōrńåmêńtš: is there a cost?

**DOI:** 10.3389/fpsyg.2023.1168471

**Published:** 2023-04-27

**Authors:** Jon Andoni Duñabeitia, Manuel Perea, Melanie Labusch

**Affiliations:** ^1^Centro de Investigación Nebrija en Cognición (CINC), Universidad Antonio de Nebrija, Madrid, Spain; ^2^Department of Languages and Culture, The Arctic University of Norway, Tromsø, Norway; ^3^Departamento de Metodología and ERI-Lectura, Universitat de València, Valencia, Spain

**Keywords:** word recognition, diacritics, word superiority effect, reading, text clarity

## Abstract

**Introduction:**

Recent research has reported that adding non-existent diacritical marks to a word produces a minimal reading cost compared to the intact word. Here we examined whether this minimal reading cost is due to: (1) the resilience of letter detectors to the perceptual noise (i.e., the cost should be small and comparable for words and nonwords) or (2) top-down lexical processes that normalize the percept for words (i.e., the cost would be larger for nonwords).

**Methods:**

We designed a letter detection experiment in which a target stimulus (either a word or a nonword) was presented intact or with extra non-existent diacritics [e.g., *amigo* (friend) vs. *ãmîgô*; *agimo* vs. *ãgîmô*]. Participants had to decide which of two letters was in the stimulus (e.g., A vs. U).

**Results:**

Although the task involved lexical processing, with responses being faster and more accurate for words compared to nonwords, we found only a minimal advantage in error rates for intact stimuli versus those with non-existent diacritics. This advantage was similar for both words and nonwords.

**Discussion:**

The letter detectors in the word recognition system appear to be resilient to non-existent diacritics without the need for feedback from higher levels of processing.

## 1. Introduction

According to leading neurally-inspired models of letter and visual-word recognition, the cognitive system develops specialized local combination detectors of increasing complexity and invariance along the left occipital cortex as a result of reading experience (Dehaene et al., 2005; Grainger et al., 2008). These detectors are arranged in a hierarchy such that lower layers respond to perceptual elements of the letters (e.g., whether a letter contains a straight line), while higher layers respond to abstract representations of letters (e.g., responding to “o,” “*o*,” and “O,” but not to “c” or “G”), and to letter combinations (e.g., frequent sequences like “ing”). Dehaene and Cohen (2007) note that these detectors can be resistant to small changes in the form of visually presented words, allowing us to read with ease CAPTCHAs (Hannagan et al., 2012), handwritten words (Barnhart and Goldinger, 2010; Vergara-Martínez et al., 2021), “leet” words (e.g., M4T3R14L, Perea et al., 2008a), and words with rotated letters (e.g., Kim and Straková, 2012; Fernández-López et al., 2023).

It is worth noting that these neurally-inspired models were originally proposed for the English orthography and did not consider the representation of diacritical letters. However, many alphabetic orthographies using the Latin script contain diacritical letters (see Protopapas and Gerakaki, 2009; Chetail and Boursain, 2019; Perea et al., 2020; Labusch et al., 2022). Theorists have suggested that diacritical letters activate their own abstract letter representations, particularly when mapping onto different phonemes than their base letters. This means that the diacritics would not be considered add-ons to their base letters but distinct letter units (Ans et al., 1998; Hutzler et al., 2004). For example, the letter “ä” in German would activate different abstract detectors than the letter “a” (Perea et al., 2022), and similar evidence has been found for diacritical consonants in Spanish (Marcet et al., 2020).

In order to gain a more comprehensive understanding of the visual-word recognition process, it is important to investigate the effects of diacritics in word identification. In the present study, we aim to explore the impact of adding non-existent diacritics to Spanish words, as in the case of “vâlïuṁ.” As Spanish readers do not possess abstract letter representations for diacritical marks such as “â,” “ï,” or “ṁ,” it is reasonable to assume that each constituent letter would enable recognition of their base letters with relatively little difficulty (e.g., “câsâ” would be processed as an allographic representation of “casa,” the Spanish word for house). Interestingly, this strategy of adding non-existent diacritics to words is commonly employed by scammers to evade spam filters on the internet when advertising products like “vâlïuṁ” on platforms that are not pharmaceutical in nature (Jáñez-Martino et al., 2022). Although this strategy may present challenges for automated filters, internet users appear to be able to read the modified words with relative ease. Thus, our study can provide insights into the underlying mechanisms of visual-word recognition and may also have practical applications in enhancing the effectiveness of automated filters.

Only a few studies have recently investigated whether there is a cost associated with the addition of non-existent diacritics to words. Labusch et al. (2023) conducted a semantic categorization task on non-diacritical French words [e.g., chēval vs. cheval (horse); the macron diacritic in ē does not exist in French] and found a small advantage of intact words (around 11 ms) over those with an additional non-existent diacritic. Furthermore, in a masked priming lexical decision experiment in English, Perea et al. (2023a) found that the recognition of a target word such as CLOCK was 7 ms faster when the identity prime was intact (e.g., clock) than when the identity prime had an additional diacritic (e.g., clóck). While these two studies demonstrated a small but consistent reading cost caused by adding a redundant diacritic to a word in tasks requiring lexical-semantic access, little is known about the mechanisms that confer resilience to changes in the visual input. Since these experiments focused on word stimuli, they cannot inform us whether it is the resistance of letter levels to distortion or whether some lexical-level feedback that normalizes percepts is responsible for the small reading cost with distorted stimuli. In other words, while there may be a small cost associated with the regularization of incorrectly marked words, it is still to be seen whether this effect spills over to other word identification processes.

To test whether the resilience of the word recognition system to visual distortion, via extra non-existent diacritics, is due to the resistance of letter detectors to visual noise or to top-down feedback, we directly compared the performance to words (i.e., letter strings with a representation at the lexical level) and nonwords. The logic is that if the cost of adding extra diacritics occurs at an early prelexical level common to words and nonwords, one would expect a similar reading cost regardless of lexicality. In this scenario, the Local Combinations Detector (LCD) model of visual-word recognition proposed by Dehaene et al. (2005) assumes that the layers of neurons in each level are resilient to variations in the visual input without requiring feedback from higher levels of processing (see Dehaene and Cohen, 2007). Thus, the LCD model would predict an equivalent, small reading cost for both words and nonwords with the extra diacritics (i.e., the locus of the reading cost would take place at a prelexical level). That is, ãmîgô hinders the processing of the Spanish word amigo (friend) in the same way that ãgîmô would hinder processing of the pseudoword agimo. An alternative explanation is that top-down lexical feedback may regularize the altered words (see Jacobs et al., 1995; Barnhart and Goldinger, 2010, for evidence of top-down lexical effects during visual word recognition). In this case, the reading cost should be smaller for words than nonwords. In this latter scenario, the cost due to the inclusion of additional diacritics would be smaller for words, since they have lexical representations that may stabilize the mental representation of the stimuli (ãmîgô vs. amigo smaller than ãgîmô vs. agimo).

In the present experiment, we chose a letter detection task to have a comparable setup for words and nonwords. This is a task that requires the same responses to words and nonwords, while being heavily influenced by top-down lexical effects. For instance, many experiments have shown that it is easier to recognize letters when embedded in words than in nonwords (i.e., a word superiority effect; see also Reicher, 1969; Wheeler, 1970; McClelland, 1976; Prinzmetal, 1992; Grainger et al., 2003; Casaponsa and Duñabeitia, 2016; see Cattell, 1886, for the first demonstration). In the task, we presented each item briefly either intact (without diacritics) or with extra non-existent diacritical marks in the target language (Spanish) [e.g., words: amigo (friend) vs. ãmîgô; nonwords: agimo vs. ãgîmô].

Our predictions for the experiment are straightforward. Firstly, we expect to observe a word superiority effect where responses are faster and less error-prone when the letters are embedded in words as compared to nonwords. This outcome would replicate earlier research findings. Secondly, if the normalization of the non-existent diacritical letters occurs at an early prelexical stage as per the LCD model (Dehaene et al., 2005), we anticipate a small reading cost for diacritical items, irrespective of whether they are presented as words or nonwords. Alternatively, if top-down lexical feedback helps normalize the encoding of non-existent diacritics as their base letters, we expect a greater reading cost for the extra diacritics to nonwords as compared to words.

## 2. Materials and methods

### 2.1. Participants

Forty four students from the Universidad Nebrija took part in this experiment. This sample size allowed us to collect 3,960 observations in each experimental condition, thus providing the appropriate power to detect small-sized effects (see Brysbaert and Stevens, 2018). The participants’ mean age was 28 years (*SD* = 8.57), and 21 self-identified as female. They were native Spanish speakers with normal/corrected-to-normal vision and gave their informed consent before the experiment. The Ethics Committee of the Universidad Nebrija approved the study protocol.

### 2.2. Procedure

**Figure 1:**
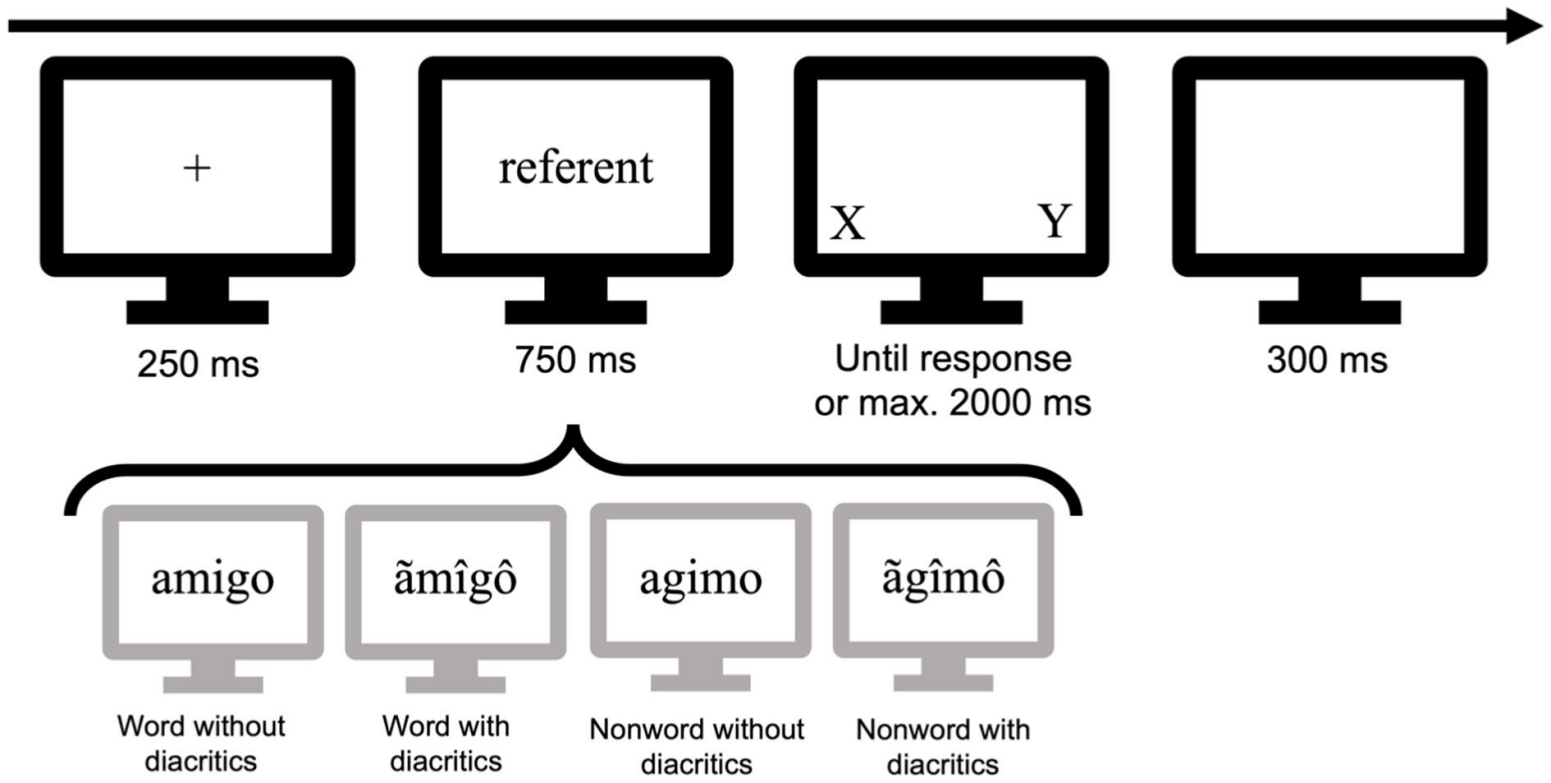
Depiction of the procedure and the four conditions used in the experiment.

The experiment was designed using Gorilla (Anwyl-Irvine et al., 2020) and the same online software was used to collect the data. Stimuli were presented in 6.3-point Times New Roman black letters on a white background. Each trial started with the centered presentation of a fixation cross (e.g., +) displayed for 250 ms that was immediately replaced with the referent word or nonword presented in lowercase for 750 ms. After this time, the string disappeared, and the two alternative letters (target and foil) were presented in uppercase on the right and left sides of the screen for 2000 ms or until a response was given. The inter-trial interval was 300 ms (see Figure 1 for a visual depiction of the procedure). Each participant was presented with a total of 360 items, in random order, and preceded by a short practice phase. There was a short rest after 180 trials. Participants were asked to respond by pressing J on the keyboard when the letter previously embedded in the string appeared on the right side of the screen and by pressing F when the correct letter appeared on the left side. The experiment lasted approximately 15 min.

### 2.3. Materials

We selected 180 Spanish 5-letter non-diacritical words [e.g., amigo (friend)] from the EsPal database (Duchon et al., 2013) with a mean Zipf frequency of 4.33 (range: 2.87–5.42). We created 180 nonwords by transposing the second and fourth letters of each base words (e.g., agimo), thus creating nonword stimuli that resembled words (see Perea et al., 2008b; see also Mirault and Grainger, 2021; Perea et al., 2023b, for recent evidence of transposed-letter effects). None of the items had repeated or diacritical letters. For each item, a new version in which at least 3 out of the 5 letters included extra non-existent diacritics (e.g., ãmîgô for amigo, and ãgîmô for agimo)—none of these diacritics exist in Spanish. The percentage of diacritical letters in each letter string was 66.89% (*SD* = 9.99) and this percentage was the same for word and nonword strings. The percentage of letters changed per string that include at least one Spanish-like diacritic [e.g., the diacritic ´ exists in Spanish vowels, but not on letters like s (ś)] was 41.38% (*SD* = 26.70). Overall, the percentage of Spanish-like diacritics used (compared to fully foreign diacritics, such as those of å or č) was 41.20%. For each string, one of its five letters was selected as the correct target for the letter detection task, and the alternative letter given in the two-alternative forced-choice procedure was never part of the string (e.g., the letters A and U for the word amigo). The test positions within the strings and the location of the presentation of the alternative letters (left/right on the screen) were balanced across items. Two experimental lists were created, and items were distributed across lists so that the same item would never appear with and without diacritic marks in the same list (see Figure 1 for a depiction of the four conditions). Each list included 90 non-diacritical words, 90 non-diacritical nonwords, 90 words with non-existent diacritics, and 90 nonwords with non-existent diacritics. Participants were assigned randomly to one of the two lists.

## 3. Results

Responses with response times below 250 ms and incorrect responses were excluded from the latency analyses (because of the 2-s deadline, responses could not be longer than 2,000 ms.) Mean latencies for correct responses and error rates are presented in Table 1. We conducted separate Bayesian Linear Mixed-Effects Models on the RT and accuracy data with the brms package (Bürkner, 2016) in R (R Core Team, 2022). The fixed factors were Lexicality (word, nonword; −0.5 and 0.5) and Diacritic Markedness (without diacritics, with diacritics; −0.5 and 0.5). Following Barr et al. (2013), we employed the models with maximal random-effect structure:

**Table 1:** Mean reaction times (in ms) and accuracy in all conditions.

	Without diacritics	With diacritics		
	Words	Nonwords	Words	Nonwords
Reaction times	690	744	688	746
Accuracy	0.96	0.937	0.947	0.922

DV ~ Lexicality*Diacritics + (1 + Lexicality*Diacritics|subject) + (1 + Diacritics|item).

The models with the RT and accuracy data were fitted with the Gaussian [via a − 1,000/RT transformation (number of responses per second)] and Bernoulli distributions, respectively. Four chains with 5,000 iterations (1,000, warm-up) were used for each model—all R̂s = 1.00. The output of the models indicates the estimate of each effect—the mean of the posterior distribution of the effect, its standard error, and its 95% Credible Interval (CrI). We interpreted evidence of an effect when the 95% CrI of its estimate did not include 0. Frequentist ANOVAs yielded the same pattern of findings as that reported here.

The reaction time analyses showed faster responses when the letters were embedded in words than in nonwords (687 vs. 745 ms; i.e., a word superiority effect; *b* = 0.12, *SE* = 0.02, 95%CrI[0.08, 0.17]). Notably, there was no evidence of an effect of Diacritic Markedness (717 vs. 717 ms, for the items with and without diacritics, *b* = 0.02, *SE* = 0.02, 95%CrI[−0.01, 0.06]) or an interaction between the two factors (*b* = −0.02, *SE* = 0.01, 95%CrI[−0.05, 0.00]).

The accuracy analysis also revealed a word superiority effect (*b* = −0.49, *SE* = 0.13, 95%CrI[−0.74, −0.23]), with letters embedded in words being recognized more accurately than letters embedded in nonwords. Additionally, letters embedded in strings with non-existent diacritical marks were recognized less accurately than those embedded in diacritic-free strings (*b* = −0.28, *SE* = 0.13, 95%CrI[−0.53, −0.02])—this effect was similar for words and nonwords (interaction: *b* = 0.13, SE = 0.16, 95%CrI[−0.19, 0.45]).

## 4. Discussion

In the present experiment, we conducted a letter search task using both word and nonword stimuli in order to investigate the potential reasons for the small reading cost associated with stimuli containing non-existent diacritics. Specifically, we sought to determine whether this cost was due to visual distortion that interfered with letter detection or to normalization through feedback from the lexical system. Participants were presented with a target stimulus that was either a non-diacritical word or a nonword. This stimulus was presented in two conditions: intact or with several non-existent diacritics [e.g., amigo (friend) vs. ãmîgô; agimo vs. ãgîmô; see Figure 1]. Following the presentation of the stimulus, a target letter and a foil (e.g., A vs. U) were presented and participants were asked to identify the target letter. Results revealed a word superiority effect, with faster and more accurate responses for target letters occurring in words compared to nonwords. Additionally, we found a small processing advantage for intact stimuli over those with extra diacritics, which was limited to accuracy and observed for both words and nonwords. No differences were observed in the letter identification times between correctly-written and altered (diacritically marked) strings.

At a theoretical level, the minimal reading cost associated with the addition of non-existent diacritics, both for words and nonwords, can be attributed to the arrays of neurons responsible for letter representations at a prelexical level, as proposed by the LCD model (Dehaene et al., 2005). According to the model, these neurons can tolerate distortions in the visual form of letters, albeit perhaps slightly less effectively than with a pristine format (see Dehaene and Cohen, 2007). However, the presented findings challenge the explanation that the reading cost of adding non-existent diacritics is due to regularization via top-down lexical effects. This account would have predicted a smaller reading cost for words than for nonwords. Therefore, the normalization effects reported in previous research with CAPTCHA words (Hannagan et al., 2012), leet words (e.g., M4T3R14L; see Perea et al., 2008a), handwritten words (Vergara-Martínez et al., 2021), or words with rotated letters (Kim and Straková, 2012) may have occurred—at least in part—at an early prelexical level, as suggested by Dehaene and Cohen (2007). In fact, research exploring the time course of these regularization effects via electroencephalographic recordings suggests that, at least for a certain type of manipulation, the visual-word recognition system is initially guided by a fast-acting pre-lexical regularization stage that is immediately followed by a lexical stage in which non-canonical representations are detected (see Duñabeitia et al., 2011). It is important to note that this interpretation is compatible with the intervention of top-down lexical processes in scenarios where the printed stimulus is heavily distorted, such as bad handwriting (Barnhart and Goldinger, 2010; Qiao et al., 2010; Vergara-Martínez et al., 2021).

Another distinctive feature of the present experiment is the addition of non-existent diacritical marks that did not provide linguistic information. These diacritical marks entail a perceptual disturbance without conflicting linguistic information. Under these circumstances, we observed only a minimal cost for words with additional diacritical marks relative to the words without diacritics. The current findings are not only of theoretical importance but also provide valuable guidelines when setting up language filters on the Internet. We have shown that words with several non-existent diacritics are processed nearly as well as their non-diacritic counterparts. Therefore, automatized language filters in chats or forums that detect inappropriate language should consider that words with non-existent diacritics in the language can be easily misread as the original words. These filters should develop detection routines that can capture these, and other regularization strategies automatically used by the human visual word recognition system. After all, humans are equally prone to buy chocolate, CH0C0L4T3, ćhõčölätē or chocolate if they like it. Another potential area for further investigation based on the present research is whether the spacing between a glyph and its corresponding diacritic is optimized for efficient reading. It is worth noting that these distances are typically determined by font designers without empirical evidence regarding their impact on lexical access (see Slattery et al., 2016, for evidence on optimal inter-letter and inter-word spacing during reading).

In summary, the present study revealed that the processing of both words and nonwords is hardly affected by adding non-existent diacritical marks. These findings point toward a hierarchical, pre-lexical processing of letters that is resilient to variations of the visual input (e.g., Dehaene et al., 2005). On the applied side, we have shown words like ćhõčölätē are processed remarkably similar to chocolate, and this must be carefully considered when implementing spam filters on the Internet.

## Data availability statement

The datasets presented in this study can be found in online repositories. The names of the repository/repositories and accession number(s) can be at: https://osf.io/sjdmw/?view_only=760f88a5b39d4ddfa12741cf702059f9.

## Ethics statement

The studies involving human participants were reviewed and approved by the Comité de Ética - Universidad Nebrija. The participants provided their written informed consent to participate in this study.

## Author contributions

JD, MP, and ML contributed to the initial conception and design of the study and wrote the first draft of the manuscript. JD conducted the experiment. JD and MP performed the statistical analyses. All authors contributed to the article and approved the submitted version.

## Funding

This study was supported by the Spanish Ministry of Science and Innovation (PID2020-116740GB-I00 and PID2021-126884NB-I00 funded by the MCIN/AEI/10.13039/501100011033), the Department of Innovation, Universities, Science, and Digital Society of the Valencian Government (CIAICO/2021/172), and the grant ISERI from the “Ayudas Fundación BBVA a Proyectos de Investigación Científica 2021.”

## Conflict of interest

The authors declare that the research was conducted in the absence of any commercial or financial relationships that could be construed as a potential conflict of interest.

## Publisher’s note

All claims expressed in this article are solely those of the authors and do not necessarily represent those of their affiliated organizations, or those of the publisher, the editors and the reviewers. Any product that may be evaluated in this article, or claim that may be made by its manufacturer, is not guaranteed or endorsed by the publisher.
